# Behaviour of Polymer Filled Composites for Novel Polymer Railway Sleepers

**DOI:** 10.3390/polym13081324

**Published:** 2021-04-18

**Authors:** Wahid Ferdous, Allan Manalo, Choman Salih, Peng Yu, Rajab Abousnina, Tom Heyer, Peter Schubel

**Affiliations:** 1 Centre for Future Materials (CFM), University of Southern Queensland, Toowoomba, QLD 4350, Australia; Allan.Manalo@usq.edu.au (A.M.); Choman.Salih@usq.edu.au (C.S.); Peng.Yu@usq.edu.au (P.Y.); Peter.Schubel@usq.edu.au (P.S.); 2 School of Engineering, Macquarie University, Macquarie Park, NSW 2113, Australia; rajab.abousnina@mq.edu.au; 3 Austrak Pty. Ltd., Brisbane, QLD 4001, Australia; Tom.Heyer@vossloh.com

**Keywords:** composite sleeper, timber replacement sleeper, GFRP, structural performance, sustainable development

## Abstract

A novel concept of polymer railway sleeper is proposed in this study that has the potential to meet static performance requirements within the cost of hardwood timber. The existing challenges of composite sleepers, such as low performance or high cost, can be overcome using this innovative concept. Such a proclamation is proven through limit state design criteria and a series of experimentations. Results show that polyurethane foam as an infill material can provide sufficient strength and stiffness properties to the sleeper, but the inadequate screw holding capacity could be a problem. This limitation, however, can be overcome using a particulate filled resin system. The findings of this study will help the railway industry to develop a timber replacement sleeper.

## 1. Introduction

The global railway industry is looking for an alternative material railway sleeper that can replace traditional timber sleeper which is suffering from premature deterioration [1,2]. To take this as an opportunity, a number of research institutions and railway sleeper manufacturing companies in different parts of the world are developing innovative polymer-based technologies. These alternative sleepers are mainly developed from recycled plastics and fibre reinforced synthetic foam materials. Recycled plastic sleepers are made of waste tyres, plastic bottles and other similar materials which is highly beneficial from the environmental viewpoint [3]. Moreover, this type of sleeper can be manufactured within the cost of hardwood timber due to the use of high volume of waste materials. However, they are suffering from low pull-out resistance, low stiffness, high thermal expansion causing plastic deformation and subsequently loosening of fasteners, low fire resistance and poor dimensional stability at service temperatures [4]. On the other hand, the Fibre-reinforced Foamed Urethane (FFU) sleeper has very similar mechanical properties but superior durability than traditional timber sleepers [5,6,7]. However, their high cost (around 5–10 times more expensive than standard timber sleepers [8]), limited shear strength due to the absence of transverse fibres and increasing concern for occupational health, safety, and environment (OHSE) [7] due to the generation of polyurethane dust during screw drilling are restricting their applications. Therefore, an alternative polymer-based material that meets both cost and performance criteria for sleeper is inevitable.

Ferdous et al. [9] investigated high performance polymer sleeper manufactured from fibre composite sandwich panels and bonded with epoxy polymer matrix. The dog bone shape of this sleeper was designed based on shape optimisation that reduced the volume of materials by up to 50% with respect to the rectangular shaped sleeper and was able to be manufactured within target price range. A similar approach for shape optimisation was considered for KLP (Kunststof Lankhorst Product) plastic sleepers which reduced the volume of plastics by 35% compared with rectangular shaped solid sleeper [10]. However, the railway industry prefers to replace timber sleeper by an alternative sleeper that looks alike timber sleepers, i.e., the rectangular shaped sleeper is the preferred choice [11]. The reason could be the rectangular shaped sleeper is easier to push into the ballast bed during spot replacement (i.e., track maintenance work). Therefore, the key scientific research challenge is how to develop an alternative material sleeper that will be rectangular in shape and perform satisfactorily within the target price range. To achieve this goal, the authors recently proposed three new concepts of internally reinforced rectangular shaped composite sleepers [4]. However, the manufacturing process of internally reinforced sleepers is complex and time consuming. Therefore, this study proposed a design concept for an externally reinforced polymer railway sleeper to address the scientific challenge. Moreover, a higher strength and stiffness can be achieved if the reinforcement is applied externally. The outcome of this study will guide sleeper manufacturers to design a polymer railway sleeper that is cheaper and faster in construction.

## 2. Development of the Sleeper Concept and Performance Evaluation

### 2.1. Selection of Suitable Polymer

The commonly used polymeric resins for structural applications are epoxy, polyester, vinyl ester, phenolic, polyurethane foam and some others. Several classes of synthetic resins manufactured by the esterification of organic compounds are available. The advantage and disadvantage of different resins are summarised in Table 1 to justify the selection of the most suitable one for manufacturing railway sleepers. One of the major considerations is the cost of resin which limits the choice among polyester, phenolic and polyurethane foam. The quick and ease of application of resin for large scale sleeper manufacturing is an essential requirement. Since the resin will be used as an infill material, moisture and UV are not of major concern. Considering this fact, polyurethane foam was selected for this study.

### 2.2. Design of PU Foam Core FRP Sleeper (Concept-1)

**Figure 1:**
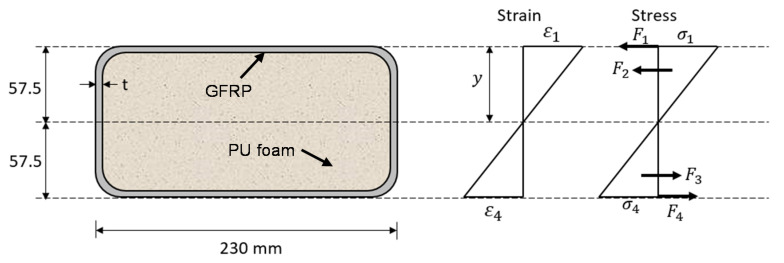
Sectional analysis of sleeper Concept-1 (ignoring the contribution of low strength infill material thus a linear strain and stress distribution for exterior Fiber Reinforced Polymer (FRP)).

This concept of sleeper is based on a glass fiber reinforced polymer (GFRP) rectangular hollow pultruded section filled with polyurethane (PU) foam as shown in Figure 1. Inspired by the sleeper concept developed on the geopolymer concrete filled GFRP tubes [13], the proposed concept is advantageous from screw drilling perspectives. While geopolymer concrete is non-drillable, the PU foam core allows on-site drilling which is an important criterion for spot replacement of deteriorated sleepers. Moreover, the exterior polymer coated GFRP offers better thermal and fire resistance compare to recycled plastics which was identified a weakness for recycled plastic sleepers. The cost of the GFRP profile is dependent on the wall thickness. To determine the minimum required wall thickness, the sectional analysis is conducted (Figure 1). It is worth noting that the contribution of PU core is ignored in the analysis due to its low strength. The strain and stress profiles are plotted in Figure 1 where the symbols have their usual meanings. Table 2 listed the properties of GFRP laminates fabricated with ten layers of fibres oriented in longitudinal (60%) and 45-degree diagonal (40%) directions with a fibre volume fraction of 55%.

The increase of sectional modulus and moment capacity with the increase of tube thickness from 1 mm to 10 mm are summarised in Table 3. The moment capacity and modulus of elasticity (MOE) of the composite sleepers can be as low as 25 kN-m [4] and 4 GPa [14], respectively. Based on the compressive failure at the top side of GFRP, Table 3 suggested that a thin layer of GFRP tube (even 1 mm) can meet the requirements of moment capacity, however, a minimum thickness of 4 mm is required to meet the target MOE of 4 GPa.

### 2.3. Materials and Manufacturing of Sleeper Concept-1

#### 2.3.1. PU Foam and GFRP Laminates

**Figure 2:**
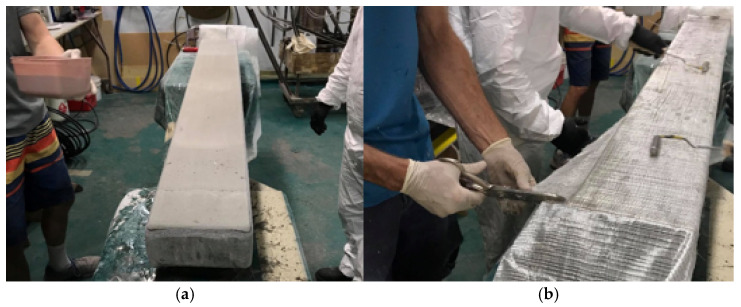
Manufacturing process. (**a**) Prefabricated PU foam core. (**b**) Applying GFRP fabrics on PU core.

A solid component made with polyurethane (PU) based resin was used as a core material of sleeper (Figure 2a). The typical compressive strength and compressive modulus of PU foam are 1 MPa and 16 MPa, respectively [15]. Two types of GFRP fabrics such as uniaxial (0 direction and 600 gsm) and triaxial (0/+45/−45 directional fibres and 823 gsm) were used to fabricate outer layer of the core. Two layers (first and last layers) of non-structural chopped strand mat (CSM) fibreglass were used together with GFRP fabrics. The very first layer of CSM was provided to ensure sufficient resin to the main GFRP layer and better bonding. The final layer of CSM was provided to avoid the drop of resin due to gravity and also for cosmetic elegance to the sleeper. The fibre stacking sequence was CSM/U/T/U/U/U/T/U/CSM, where U and T represents unidirectional and triaxial fabrics, respectively. The lamination process was done at the Buchanan Advanced Composites (BAC) in Toowoomba.

#### 2.3.2. Manufacturing Method

The PU core was prepared in a closed mould before applying laminates. The PU core was wrapped with GFRP laminates using hand lamination process. The wrapping was done by two steps to avoid sagging due to gravity (a) applied on two sides and the top surface and (b) flipped over and repeat the same process (i.e., applied two sides and the other surface). The fibres were overlapped at two sides of the sleeper for full depth.

A particulate filled resin mix was prepared using polyester resin (Polyplex 1472 Infusion Resin 25, density 1.10 g/cm^3^), NOROX CHM-50 hardener (density 1.06 g/cm^3^) and fly ash with a mixing ratio of Resin: Hardener: Fly ash = 2000:40:200 g. The resin and hardener were mixed before the fly ash added to the mix. The gel time (working time) of the resin mix was around 1 h. The samples were post cured at 80 °C for 3 h. The overall casting process are shown in Figure 2. Although the GFRP laminates were applied using hand lamination process for the research purpose, the approach was used to simulate pultruded hollow rectangular profiles for large scale manufacturing.

### 2.4. Results and Discussion—Performance Evaluation of Sleeper Concept-1

#### 2.4.1. Density

The dimensions of the PU foam core sleeper were 243 mm (W) × 120 mm (D) × 2130 mm (L). The overall weight of the sleeper was 39 kg that provided an equivalent density of 640 kg/m^3^. This density is lower than the density of traditional softwood timber sleepers (855 kg/m^3^) and able to overcome the limitation of heavy weight for concrete sleepers (2000 kg/m^3^). Moreover, the density is also slightly lower than the recycled plastic sleepers (850–1150 kg/m^3^) and close to FFU (fibre-reinforced foamed urethane) synthetic sleeper (740 kg/m^3^) [10]. The lower density of the proposed concept is due to the use of lightweight foam core. The low density is preferable for ease of handling sleepers but might create track stability issue.

#### 2.4.2. Bending Modulus of Elasticity (MOE)

**Figure 3:**
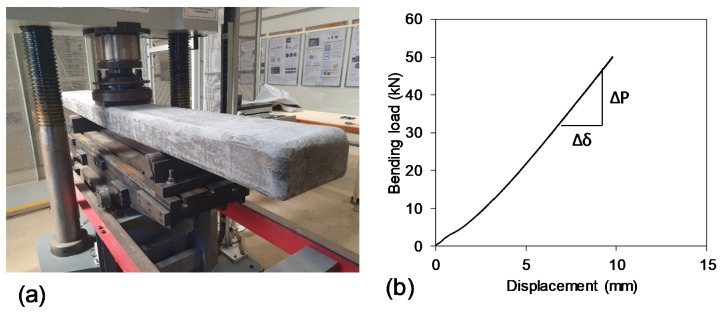
Non-destructive three-point bending test (**a**) test setup and (**b**) load-displacement behaviour.

Bending modulus of elasticity is an important property on which the deflection characteristics of sleeper are dependent. The MOE is the function of the initial slope of the load-displacement curve obtained from midspan bending test. Therefore, a non-destructive test (Figure 3a) up to 50 kN was carried out under three-point bending with a span of 1130 mm (same as narrow gauge length of sleeper).

Figure 3b plotted the load-displacement curve where it can be seen that the bending behaviour is almost linear up to 50 kN load. The effective modulus of elasticity of the sleeper was found 5.15 GPa as determined by Equation (1), where *a*, *L*, *I* and Δ*P*/Δ*δ* are shear span, span, effective moment of inertia of the sleeper, and slope of the load–displacement curve, respectively. The *MOE* for softwood timber sleeper can be as low as 7.4 GPa. Moreover, the *MOE* for recycled plastic sleeper varies between 1.5 GPa and 1.8 GPa while it is 8.1 GPa for FFU synthetic sleeper [10]. The American Railway Engineering and Maintenance-of-Way Association (AREMA) specification indicated that the *MOE* for polymer composite sleeper should be at least 1.17 GPa [16]. The incorporation of fibres in PU foam core sleeper provided higher modulus than recycled plastic materials. However, the stiffness is slightly lower than softwood timber and FFU due its lower density.

(1) $$MOE=\frac{a}{48I}\left(3L^{2}-4a^{2}\right)\left(\frac{\Delta P}{\Delta \delta }\right)$$

#### 2.4.3. Compression Modulus of Elasticity

**Figure 4:**
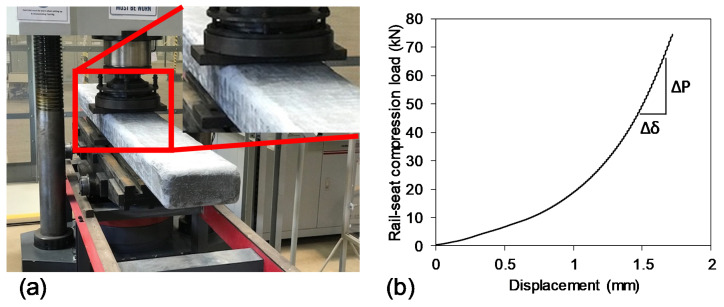
Rail-seat compression test (**a**) test setup and (**b**) load-displacement behaviour.

The compression modulus of elasticity of sleeper indicates the deformation characteristics when the sleeper is being compressed. A non-destructive rail-seat compression test was conducted to determine these properties. The compression load (Figure 4a) was applied up to 72 kN which is the design rail-seat load for narrow-gauge rail-track [9]. The sleeper was supported by two steel plates. The load was applied at mid-span due to the homogeneous property along the length of the sleeper.

The compression load-displacement behaviour is plotted in Figure 4b. The compression test provided a non-linear load-displacement curve with increasing slope. The initial slope up to 10 kN was linear and lower than other portions of the curve. This implies the specimen was locally compressed at the initial stage and the slope started to increase thereafter due to stopping local compression. Therefore, the maximum slope in the entire load-displacement curve was used to determine the compressive modulus of elasticity using Equation (2). In this equation, *D*, *A* and Δ*P*/Δ*δ* are the depth of sleeper, effective compression area and maximum slope of the load-displacement curve. The compressive modulus of elasticity was obtained 450 MPa which is higher than the same property for recycled plastic sleepers (176–269 MPa) but lower than timber sleepers (650 MPa) [17]. Understanding the compressive modulus of sleeper is important as the screw holding ability of sleeper is dependent on this property.

(2) $$MOE_{com}=\frac{D}{A}\times \frac{\Delta P}{\Delta \delta }$$

#### 2.4.4. Modulus of Rupture (MOR)

**Figure 5:**
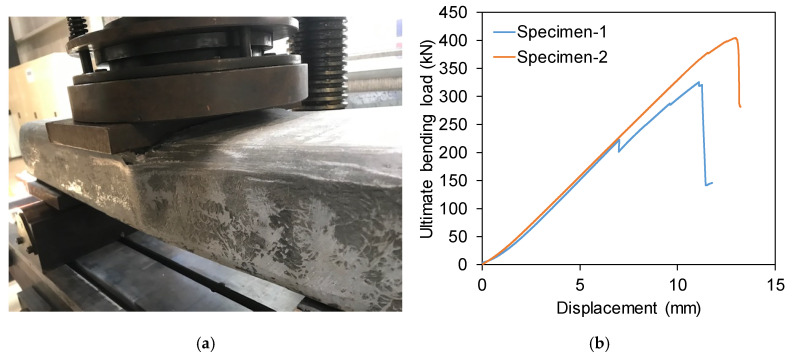
Ultimate test results. (**a**) Test setup and failure mode. (**b**) Load-displacement behaviour.

Modulus of rupture measures the load carrying capacity of sleeper in bending. The test was conducted following the procedure described in the AS 1085.22 [18]. Two specimens were tested under centre point bending at a span of 600 mm until the failure (Figure 5a). It was observed that the specimens were failed in skin buckling and compression. The specimens failed in skin buckling due to the discontinuation of fibres in the hand lamination manufacturing process. This could be avoided if the GFRP rectangular hollow profile is made in a pultrusion process. The maximum load and displacement (Figure 5b) observed from calibrated equipment were 325 kN at 11.1 mm for specimen-1 and 405 kN at 13 mm for specimen-2, respectively, provided an average *MOR* of 94 MPa according to Equation (3) where *M* is the average ultimate moment and *c* is the distance of outer fibre from the neutral axis. The *MOR* obtained for this sleeper is significantly higher than the *MOR* of softwood timber (22–34 MPa) and hardwood timber sleepers (55 MPa) [4].

(3) $$MOR=\frac{Mc}{I}$$

#### 2.4.5. Pull-Out Resistance

**Figure 6:**
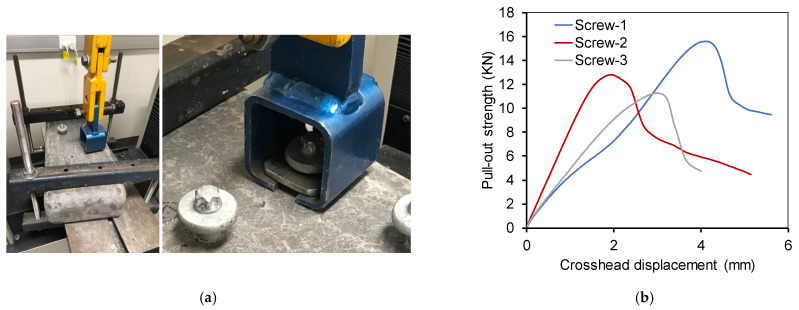
Pull-out behaviour. (**a**) Pull-out test setup. (**b**) Pull-out force vs displacement.

Pull-out resistance measures the screw holding capacity of sleeper. This is an important property that ensures rail alignment in correct gauge. The pull-out resistance is determined based on the force required to pull-out the screw. Three standard 16 × 105 rail screws (shank diameter 16 mm, underhead length 105 mm and shank length 35 mm) supplied by Cold Forge Pty Ltd (Caringbah, NSW, Australia) were inserted into the sleeper. The test setup is shown in Figure 6a and the pull-out force and crosshead displacement is plotted in Figure 6b. The maximum pull-out resistance provided by three screws were 15.57 kN, 12.78 kN, and 11.29 kN with an average of 13.2 kN (standard deviation 2.17). This resistance is lower than the minimum pull-out resistance of 22.2 kN required as per AREMA specification [16]. Moreover, the resistance is also significantly lower than the 40 kN pull-out resistance required for traditional timber sleeper as per AS1085.18 standard [19]. The low pull-out resistance is due to the low transverse shear capacity of the PU core.

### 2.5. Findings from Sleeper Concept-1

It is obvious that the alternative sleeper has to perform satisfactorily as per standard requirements. The performance of the sleeper Concept-1 obtained from the extensive testing program is tabulated in Table 4. It can be seen that the bending modulus of elasticity and modulus of rupture of the PU foam core FRP sleeper (Concept-1) satisfactorily meet the performance criteria while density and rail-seat compression modulus are slightly lower than timber. However, the screw pull-out resistance is lower than the minimum requirement as per AREMA specification for polymer composite sleeper. Therefore, a further investigation is necessary to overcome the limitations of PU foam core FRP sleeper. The next section highlighted how to improve screw pull-out resistance without sacrificing other requirements.

## 3. Overcoming Challenges of Sleeper Concept-1

### 3.1. Development of PFR Core FRP Sleeper Concept (Concept-2)

The low strength capacity of PU foam core in sleeper Concept-1 is the main issue identified for low screw holding capacity as no crack was observed on the FRP. Therefore, the strength properties of core material need to be improved. One approach to improve the core properties is to use the particulate filled resin (PFR) system. Ferdous et al. and Khotbehsara et al. [20,21,22] developed a PFR that has superior strength properties and could be suitable for manufacturing polymer railway sleepers. However, the PFR is expensive compared to PU core since the latter one is a foam that can add volume without increasing cost. Therefore, a minimum volume of PFR should be used to minimise the cost of sleeper. Since the purpose of introducing PFR is to improve the screw holding capacity, they can be filled at the rail-seat region only while the other parts of the hollow FRP tube can be filled with low cost material such as ordinary portland cement (OPC) concrete which is even cheaper than PU foam core. This approach may increase the overall weight of the sleeper as both PFR and OPC concrete are heavier than PU core. However, the density of sleeper Concept-1 is lower than timber (Table 4) and thus a further increase of density may not create problem. Moreover, it is expected that the density of timber replacement sleeper should be similar to the density of timber. While lighter sleepers are easy to handle, they are however less effective to provide lateral stability of the rail-track. A schematic diagram of the whole concept is provided latter.

### 3.2. Materials and Manufacturing of Sleeper Concept-2

The 5.2 mm thick FRP tubes used in Concept-2 were fabricated by pultrusion process and were made of E-glass fibre and vinyl-ester resin with a fibre volume fraction of 60%. The hollow pultruded section is a preferred choice over the laminates overlapping method discussed in Concept-1 as the latter one was failed due to skin buckling (Figure 5a). The PFR in this study was prepared by mixing polyester resin and methyl ethyl ketone peroxide (MEKP) hardener with a mixing ratio of 100:1.5 g and filled the resin matrix with short polypropylene (PP) fibres and fly ash fillers. The fillers were mixed together prior to mixing resin and hardener. Once prepared a homogeneous resin and hardener mix, the mixed fillers were added to the resin system. The bottom of the FRP tubes were sealed and placed vertically to fill the tubes from the top end. When tubes were filled with OPC and PFR, the pouring was done in separate days for each mix. Before testing, the samples were cured in normal temperature and humidity for more than 28 days.

### 3.3. Results and Discussion—Performance Evaluation of Sleeper Concept-2

#### 3.3.1. Pull-Out Resistance

**Figure 7:**
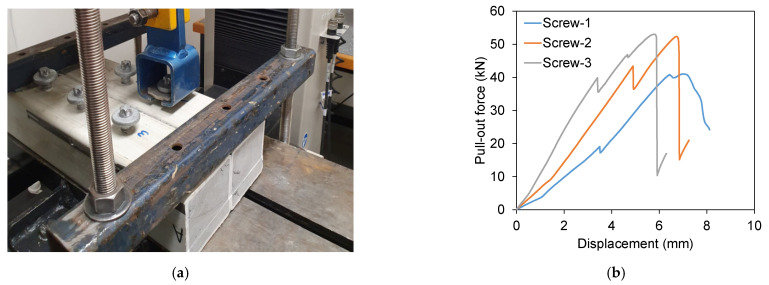
Pull-out test of the PFR core FRP sleeper (Concept-2). (**a**) Pull-out test setup. (**b**) Pull-out force vs displacement.

Six standard screws were inserted in the PFR core FRP sleeper (Concept-2) as shown in Figure 7a. The results are plotted in Figure 7b. The first major drop of load for Screw-1, Screw-2 and Screw-3 were observed at 40.47 kN, 43.20 kN and 39.81 kN with an average of 41.16 kN (standard deviation of 1.8). Only three screws were tested as the ultimate pullout resistance for the first three screws were consistent. It can be seen that the PFR core (41.16 kN) in sleeper Concept-2 significantly improved the pull-out resistance when compare to PU foam core (13.2 kN only) in sleeper Concept-1. Moreover, the average screw holding capacity of PFR core sleeper is very similar to the minimum required capacity for timber sleepers (40 kN as per AS1085.18 standard). Therefore, PFR core has potential for manufacturing polymer railway sleepers.

#### 3.3.2. Effect of the Joint between OPC and PFR

**Figure 8:**
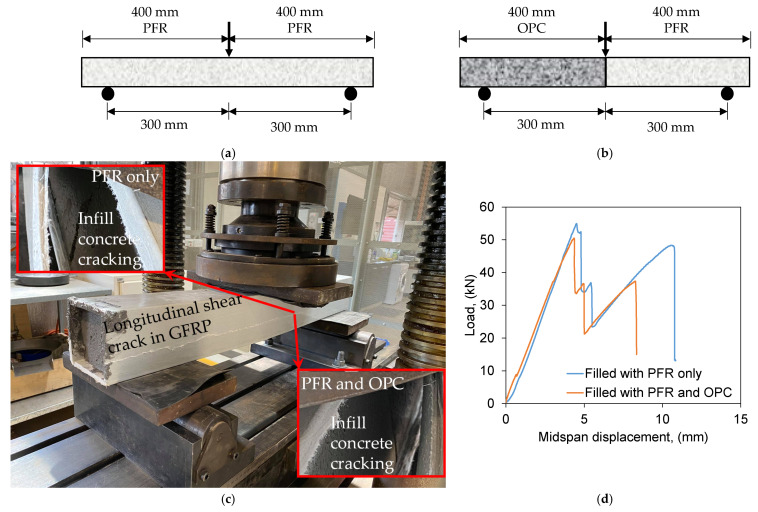
Effect of joint between OPC and PFR. (**a**) FRP tube filled with PFR only. (**b**) FRP tube filled with PFR and OPC. (**c**) Failure mode. (**d**) Load-displacement plot.

Since the primary component of drillable PFR is resin, it is more expensive than normal OPC concrete which is non-drillable. Therefore, an optimal use of PFR only at rail-seat region where the screws are drilled could significantly minimise the cost of sleepers. The other parts should be filled with normal OPC concrete. In such a case, several bond areas between PFR and OPC concrete are obvious which could create weak zones due to the variation of their elastic modulus properties. To address this concern, one of the two FRP tubes was filled with PFR and the other one was filled with a combination of normal OPC concrete and PFR by equal volume (Figure 8a,b). Centre-point bending test was conducted to create maximum moment at the bond area as shown in Figure 8b. Both beams were failed in a similar manner due to the longitudinal shear cracks appeared in FRP tubes (Figure 8c). The FRP tubes were opened after ultimate failure and it was observed that the in-fill concrete was cracked at the load point for both beams, particularly the in-fill concrete in the second beam was failed at the OPC-PFR interface (Figure 8c). However, the joint between OPC and PFR did not affect the overall behaviour as shown in Figure 8d. The load-displacement behaviour of both beams were very similar in terms of strength, stiffness and post-cracking behaviour. This is because the overall behaviour of the beam is governed by FRP tubes and the type of in-fill materials has less impact due to the confinement effect. Therefore, it can be said that the PFR is compatible with OPC as in-fill material for manufacturing polymer railway sleepers.

#### 3.3.3. Full Length Sleeper Deflection Behaviour

**Figure 9:**
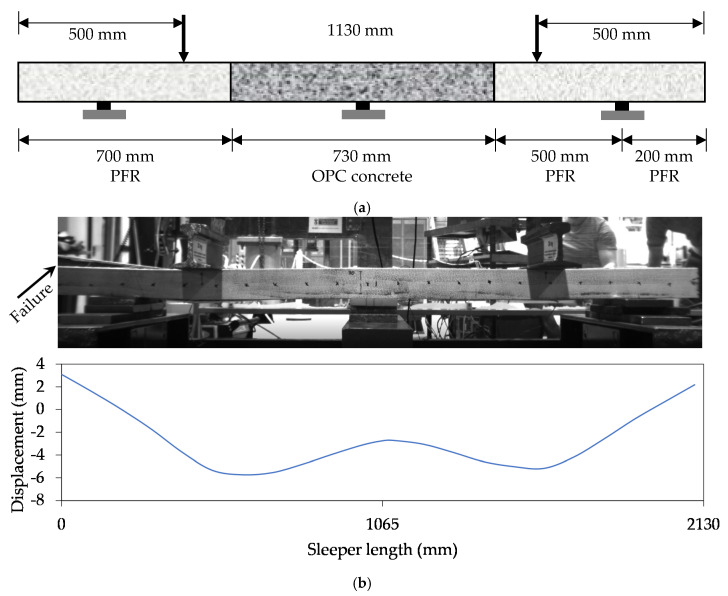
Full-scale deflection behaviour. (**a**) Five-point bending (5-PB) test setup. (**b**) Bending profile of sleeper.

There still remains the question of how the full-scale sleeper will behave in rail-track. To understand the in-track deflection behaviour of full-scale sleeper, a five-point bending test was conducted. The justification of the five-point bending test setup for railway sleeper is discussed in [4]. The load was applied until the ultimate failure at 143 kN of total load (i.e., 72 kN at each rail-seat). The specimen was failed due to the longitudinal shear crack in FRP at the upper end. The bending shape of the beam is plotted in Figure 9b. It can be seen that a positive bending (sagging moment) occurred at rail-seat region while negative bending (hogging moment) observed at mid-span. This is the generalbehaviour observed in real rail-track condition [23]. The maximum deflection observed at rail-seat location is 5.73 mm which is larger than the theoretical deflection of 2.66 mm determined in [4]. This is because a smaller section of 100 × 100 mm instead of the actual sleeper section (230 × 115 mm^2^) was tested to understand whether the concept is suitable for manufacturing sleeper. In this study, 2.15 times larger (i.e., 5.73/2.66) deflection of the beam than the deflection of the actual size sleeper is expected as the dimensions of the actual sleeper are 2.3 times wider (i.e., 230/100) and 1.15 times deeper (i.e., 115/100).

### 3.4. Findings from Sleeper Concept-2

**Figure 10:**
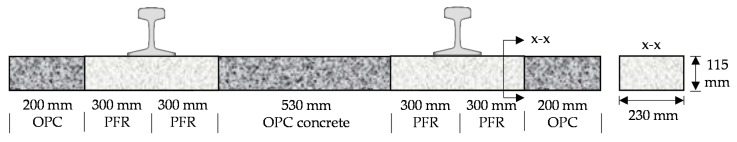
Proposed final design of polymer sleeper (based on Concept-2).

Sleeper Concept-2 was introduced to overcome the challenge of low screw holding capacity in sleeper Concept-1. Replacing PU foam core by PFR can significantly improve and meet the requirements of pull-out resistance for polymer sleepers. Since PFR is an expensive material, its optimal use is important to minimise the cost of sleepers. Therefore, the rail-seats region of the FRP tube need to be filled with drillable PFR while other parts can be filled with non-drillable OPC concrete. It was found that the joint between PFR and OPC concrete does not affect the overall behaviour. Figure 10 proposed the distribution of infill materials along the sleeper length. Only 600 mm at the two rail-seat locations need PFR as an infill material based on the stress distribution pattern described in [9]. This concept can save almost 50% volume of PFR. Moreover, the FRP tube with a thickness of 4 mm can provide sufficient strength and stiffness properties. It is estimated that the materials cost of the proposed sleeper concept would be approximately AU$100 per sleeper which is quite below than the target final sleeper cost of AU$160 indicated by Queensland Rail (excluding sleeper reprocessing cost at the end of their life). Although the final product cost of $160 is twice the cost of traditional timber sleeper ($80), however, the life cycle cost of composite sleeper (50 years design life) is expected to be lower than timber sleeper (15 years design life). Therefore, manufacturing a high performance and cost effective polymer composite railway sleeper is possible using the proposed concept.

## 4. Conclusions

This study proposed a new concept for manufacturing fibre reinforced polymer railway sleeper. The systematic design approach and experimental program identified new materials and method for developing composite sleeper concept. The following conclusions are made:

- Filled FRP tube is a promising concept for developing polymer railway sleepers. To meet the strength and stiffness requirements, a minimum tube thickness of 4 mm is necessary.
- Polyurethane foam as an infill material can provide sufficient bending and compression properties. However, it cannot provide sufficient resistance to hold screws.
- Particulate filled resin (PFR) system as an infill material can overcome the limitation of low screw holding capacity that was observed in polyurethane foam.
- The expensive and drillable infill PFR material can be replaced by the inexpensive and non-drillable OPC concrete except rail-seat locations.
- The joint between PFR and OPC concrete does not affect the overall performance of the sleeper as the behaviour of the sleeper is governed by an external FRP tube and the type of in-fill materials have only minimal impact due to the confinement effect.
- The proposed design of sleeper only requires 50% volume of PFR as infill material that lead to manufacture a high performance and cost effective railway sleeper technology.

Railway sleepers are often subjected to impact and fatigue loading due to flat wheel and repeated movements of the train. Therefore, an in-depth understanding of the impact and fatigue behaviour of the polymer sleeper will ensure its suitability to replace existing timber sleepers. To understand the fatigue behaviour of a polymer sleeper, the author attempted to investigate the fatigue behaviour of GFRP laminates [24,25].

## Figures and Tables

**Table 1:** Advantage and Disadvantage of Different Resins [12].

Types of Resin	Advantage	Disadvantage
Epoxy	High mechanical and thermal properties High water resistance Long working times available High temperature resistance Low cure shrinkage	More expensive than vinyl esters Mixing is critical
Polyester	Easy to use Low cost	Limited mechanical properties High styrene emissions in open moulds Limited range of working times High cure shrinkage
Vinyl ester	Very high chemical resistance Higher mechanical properties than polyesters	Postcure required for high properties High styrene content Expensive than polyesters High cure shrinkage
Phenolic	Good mechanical properties Heat and impact resistant High chemical and moisture resistance Low cost Low smoke emission	Very low toughness Brittle nature Humidity badly affects its resistance
Polyurethane foam	Low cost High thermal insulation properties Quick and easy application Highly adhesive and extremely lightweight Does not create dust or release harmful gases	Spray foam insulation method might be expensive

**Table 2:** Nominal Properties of GFRP Laminates.

Properties	Value	Unit
Tensile failure strain	0.035	-
Tensile failure stress	425	MPa
Tensile modulus	16.5	GPa
Compressive failure strain	0.025	-
Compressive failure stress	280	MPa
Compressive modulus	11.5	GPa

**Table 3:** Increase of Sleeper Capacity with Tube Thickness.

Tube Thickness	Neutral Axis Depth	I	Moment	MOE
mm	mm	mm4	kN-m	GPa
1	61.09	29490994	89	2.01
2	61.08	29489098	175	2.73
3	61.06	29485321	258	3.41
4	61.04	29481565	339	4.07
5	61.02	29477830	416	4.70
6	61.00	29474117	491	5.30
7	60.98	29470424	563	5.86
8	60.96	29466753	633	6.40
9	60.93	29461286	699	6.92
10	60.91	29457667	763	7.41

**Table 4:** Performance Comparison of PU Foam Core FRP Sleeper (Concept-1).

Properties	Concept-1	Softwood	AREMA Requirements	Observation
Density (kg/m^3^)	640	855	Not available	Lighter than timber
Bending modulus of elasticity (GPa)	5.15	7.4	1.17	Satisfactory
Rail-seat compression modulus (MPa)	450	650	Not available	Lower than timber
Modulus of rupture (MPa)	94	22–34	13.8	Satisfactory
Screw pull-out resistance (kN)	13.2	40	22.2	Unsatisfactory

## Data Availability

Not applicable.
